# Recurrent Urinary Tract Infection in Diabetics: A Retrospective Analysis

**DOI:** 10.7759/cureus.87816

**Published:** 2025-07-13

**Authors:** Hanan Wajid, Hafsa Manzoor, Misbah Manzoor, Sabeen Mirza, Hassaan Saeed Khan, Shahzaib Hassan, Bilawal Ali

**Affiliations:** 1 Internal Medicine, Gondal Medical Complex, Gujranwala, PAK; 2 Pharmacology, Abu Umara Medical and Dental College, Lahore, PAK; 3 Internal Medicine, Fatima Memorial Hospital, Lahore, PAK; 4 Internal Medicine, Avicenna Medical and Dental College, Lahore, PAK; 5 Internal Medicine, Shalamar Medical & Dental College, Lahore, PAK; 6 Internal Medicine, District Headquarter (DHQ) Hospital, Dera Ghazi Khan, PAK; 7 Internal Medicine, Allama Iqbal Teaching Hospital, Dera Ghazi Khan, PAK

**Keywords:** antibiotic resistance, diabetes mellitus, glycemic control, immune response, urinary tract infection

## Abstract

Background

Recurrent urinary tract infections (rUTIs) are a common complication in diabetic individuals due to impaired immune responses, poor glycemic control, and anatomical abnormalities.

Objective

Given the high burden of diabetes, rising antimicrobial resistance, and the paucity of local epidemiological data on recurrent UTIs in this population, this study aimed to determine the clinical characteristics, microbial profile, antibiotic resistance patterns, and associated risk factors of recurrent urinary tract infections in patients with diabetes mellitus.

Methods

This retrospective study was conducted at Fatima Memorial Hospital, Lahore, Pakistan from April 2023 to April 2025. A total of 320 patients with a confirmed diagnosis of diabetes and documented episodes of recurrent urinary tract infections were included in the study. Clinical and laboratory data were extracted from electronic and paper-based medical records using a standardized data collection form.

Results

The mean age of patients was 59.4 ± 11.2 years, with females comprising 218 (68.1%) of the cohort. Type 2 diabetes accounted for 286 (89.4%) of cases. Poor glycemic control (HbA1c > 7.5%) was observed in 229 (71.6%) of patients. The most common pathogen was E. coli (180, 56.3%), followed by Klebsiella pneumoniae (59, 18.4%) and Enterococcus faecalis (29, 9.1%). Multidrug resistance was noted in 146 (45.6%) of isolates; 118 (36.9%) were extended-spectrum beta-lactamases (ESBL) producers. Logistic regression identified female gender (OR: 2.84), poor glycemic control (OR: 3.12), diabetes duration >10 years (OR: 2.45), diabetic nephropathy (OR: 2.19), and prior UTI-related hospitalization (OR: 3.67) as significant predictors of recurrence and resistance (p < 0.05).

Conclusion

It is concluded that recurrent UTIs are common in diabetic patients and are significantly influenced by glycemic control, gender, disease duration, and renal complications. Rising antibiotic resistance among uropathogens necessitates routine culture-based treatment and individualized preventive strategies to improve outcomes in this high-risk group.

## Introduction

Urinary tract infections are among the most frequently encountered bacterial infections worldwide, affecting millions annually across all age groups and demographics. Urinary tract infections (UTIs) are exacerbated in diabetic individuals due to anatomical, metabolic, and immunological alterations [1]. Recurrent urinary tract infections, as defined by two or more that occurred in six months or three or more in the course of a year, are a complex group characterized by recurrent symptoms often associated with antibiotic resistance and poor response to treatment [2]. Increasing concern exists regarding the growing problem of recurrent urinary tract infections in diabetics, especially keeping in view the global spread of diabetes. The outcome of diabetes mellitus compromises various defense systems in the urinary tract [3]. Durable exposure to increased levels of glucose leads to glucosuria, thereby contributing to a suitable nutrient supply to bacteria and to their proliferation. Impaired emptying of the bladder and urinary stasis, which are triggers for frequent recurrence of infections, are also caused by diabetic autonomic neuropathy. An inherent immunodeficiency compromised by defective neutrophil chemotaxis, phagocytic capability and bactericidal competency further weakens the host’s defence mechanism against infection [4]. Besides, microvascular problems such as diabetic nephropathy and ischemia in uroepithelium may weaken tissue integrity and reduce local colonization resistance [5].

In diabetic patients, UTIs are more frequently caused by multidrug-resistant bacteria compared to the general population. Although the most common uropathogen, Escherichia coli is also being less commonly detected amongst diabetic patients in comparison with non-E. coli strains, such as Klebsiella, Enterococcus, Proteus and Pseudomonas, in particular amongst those who have recurrent uropathogen infection. Given the ubiquitous formation of biofilms coupled with their multidrug resistance, treatment becomes complicated and therefore necessitates individualized regimes of antibiotics, which are based on sensitivity results. The change in the type of pathogens and the augmented burden of extended-spectrum beta-lactamase generating bacteria make significant barriers to therapy and highlight the necessity of antimicrobial stewardship [6]. Recurrent urinary tract infections in diabetic patients have serious clinical implications beyond everyday discomfort [7]. Constant urinary tract infections, if left untreated, may ascend to involve the upper urinary tract, thus causing acute or chronic pyelonephritis. Recurrent infections are also linked with high levels of renal function deterioration among diabetics with underlying susceptibility to nephropathy. In women with diabetes, the risk of recurrent urinary tract infection is increased due to anatomical differences and changes in hormones after menopause, disrupting the vaginal environment and reducing local immunity [8].

Variations in the prevalence of recurrent urinary tract infections in diabetics depend on the population studied, diagnostic approaches, and availability of high-quality health and healthcare resources. Research shows that at least 25-30 percent of diabetics report at least one urinary tract infection each year, and many of this population suffer from repeated infections. However, research concerning South Asia and even Pakistan remains insufficient [9]. Many modifiable and non-modifiable risk factors have been identified in medical literature. Examples are poor glucose control, long-standing diabetes, and being female [10]. In resource-limited settings, decrements due to lack of proper personal cleanliness, delayed urination, and inadequate fluid consumption have a significant part in elevating the risk. Successful prevention of recurrent urinary tract infection in diabetics should include several interventions [11]. All non-antibiotic interventions (D-mannose, probiotics, cranberry extract, and topical estrogen therapy in postmenopausal women) are under research to be used as a supplementary measure, but results are inconclusive. The long-term use of prophylactic antibiotics is often unpopular due to concerns about increasing the resistance to antibiotics, adverse reactions, and alteration in the body’s beneficial bacteria constituency [12].

Objective

Given the high burden of diabetes, rising antimicrobial resistance, and the paucity of local epidemiological data on recurrent UTIs in this population, this study aimed to determine the clinical characteristics, microbial profile, antibiotic resistance patterns, and associated risk factors of recurrent urinary tract infections in patients with diabetes mellitus.

## Materials and methods

Methodology

This retrospective study was conducted at Fatima Memorial Hospital, Lahore, Pakistan from April 2023 to April 2025. A total of 320 patients with a confirmed diagnosis of diabetes and documented episodes of recurrent urinary tract infections were included in the study.

Inclusion and exclusion criteria

Participants must be patients over the age of 18 years who have been diagnosed with either type 1 or type 2 diabetes mellitus according to the American Diabetes Association (ADA) criteria. Additionally, they must have a documented history of recurrent urinary tract infections, defined as either two or more episodes within a six-month period or three or more episodes within a single year [13]. Finally, the complete medical records for these patients must be available, specifically including urine culture reports, antibiotic sensitivity patterns, and relevant laboratory findings.

Patients were excluded if they had indwelling urinary catheters at the time of study enrollment (baseline). Individuals with structural urogenital abnormalities diagnosed prior to their first urinary tract infection episode, such as vesicoureteral reflux or neurogenic bladder, were also excluded. Furthermore, pregnant women were not eligible for participation. Lastly, immunocompromised individuals, including those with HIV infection, active malignancy, or those receiving chronic immunosuppressive therapy, were excluded from the study.

Data collection

Clinical and laboratory data were extracted from electronic and paper-based medical records using a standardized data collection form. Information collected included demographic variables such as age, sex, and body mass index (BMI), along with clinical parameters including the duration of diabetes, most recent HbA1c levels as a marker of glycemic control, and the presence of comorbid conditions like hypertension and chronic kidney disease. The number and timing of UTI episodes were recorded, as well as findings from urine routine microscopy, urine culture reports, and sensitivity patterns. Additional laboratory data included fasting blood glucose, serum creatinine levels, and estimated glomerular filtration rate (eGFR). Treatment history and any prior hospitalizations related to urinary tract infections were also documented.

Statistical analysis

Data were entered and analyzed using SPSS version 26 (IBM Corp., Armonk, NY, USA). Descriptive statistics were used to summarize the baseline characteristics of the study population. Continuous variables were expressed as means and standard deviations. Categorical variables were presented as frequencies and percentages. Multivariable logistic regression was applied to determine independent predictors of recurrent urinary tract infections in diabetic patients. A p-value of less than 0.05 was considered statistically significant.

## Results

The study included 320 diabetic patients, with a mean age of 59.4 ± 11.2 years. A total of 218 patients (68.1%) were female, and 286 (89.4%) had type 2 diabetes mellitus. A diabetes duration of more than 10 years was observed in 157 patients (49.1%), while poor glycemic control (HbA1c >7.5%) was present in 229 (71.6%). Hypertension was noted in 203 patients (63.4%), and chronic kidney disease (stage ≥3) was documented in 73 patients (22.8%) (Table 1).

**Table 1:** Baseline Characteristics of the Study Population (n = 320) Data are represented as Mean ± SD or percentages (%). DM: diabetes mellitus, CKD: chronic kidney disease

Variable	Value
Age (years)	59.4 ± 11.2
Gender (Female)	218 (68.1%)
Type 2 DM	286 (89.4%)
Duration of DM >10 years	157 (49.1%)
HbA1c >7.5%	229 (71.6%)
Hypertension	203 (63.4%)
CKD (Stage ≥3)	73 (22.8%)

A total of 187 patients (58.4%) experienced three or more urinary tract infection episodes per year, while 133 (41.6%) had two episodes within six months. Regarding the microbial profile, Escherichia coli (E. coli) was the most frequently isolated pathogen, identified in 180 cases (56.3%). Other organisms included Klebsiella pneumoniae in 59 (18.4%), Enterococcus faecalis in 29 (9.1%), Pseudomonas aeruginosa in 21 (6.6%), and other pathogens such as Proteus species and Candida in 31 cases (9.6%) (Table 2).

**Table 2:** Frequency of Recurrent UTI Episodes Among Diabetic Patients UTI: urinary tract infection

Recurrent UTI Pattern	Number of Patients	Percentage
≥3 episodes/year	187	58.4%
2 episodes in 6 months	133	41.6%
Microbial Profile of Isolates from Recurrent UTIs		
Escherichia coli	180	56.3%
Klebsiella pneumoniae	59	18.4%
Enterococcus faecalis	29	9.1%
Pseudomonas aeruginosa	21	6.6%
Others (Proteus, Candida)	31	9.6%

Antibiotic susceptibility testing revealed that E. coli isolates showed the highest sensitivity to nitrofurantoin at 78.2%, followed by fosfomycin at 71.6%. In contrast, ciprofloxacin demonstrated poor sensitivity, with only 36.2% of isolates susceptible and a high resistance rate of 63.8%. Regarding resistance patterns, 146 isolates (45.6%) were identified as multidrug-resistant organisms (MDROs), and 118 (36.9%) were extended-spectrum beta-lactamase (ESBL) producers, indicating a significant burden of antimicrobial resistance among recurrent UTI pathogens in diabetic patients (Table 3).

**Table 3:** Antibiotic Susceptibility Pattern of Escherichia coli ESBL: extended-spectrum beta-lactamases

Antibiotic	Sensitivity (%)	Resistance (%)
Nitrofurantoin	78.2	21.8
Fosfomycin	71.6	28.4
Ciprofloxacin	36.2	63.8
Resistance Type		
Multidrug-resistant organisms (MDRO)	n=146	45.6%
ESBL-producing organisms	n=118	36.9%

Logistic regression analysis revealed that female gender (OR: 2.84, 95% CI: 1.72-4.69, χ² = 15.27, p < 0.001) and poor glycemic control with HbA1c >7.5% (OR: 3.12, 95% CI: 1.98-4.91, χ² = 18.63, p < 0.001) were significantly associated with recurrent UTIs. A diabetes duration of more than 10 years also posed a higher risk (OR: 2.45, 95% CI: 1.53-3.93, χ² = 12.11, p = 0.002), as did the presence of diabetic nephropathy (OR: 2.19, 95% CI: 1.22-3.93, χ² = 6.59, p = 0.01). Prior hospitalization for UTI showed the strongest association with recurrence (OR: 3.67, 95% CI: 1.90-7.06, χ² = 20.74, p < 0.001) (Table 4).

**Table 4:** Logistic Regression Analysis of Risk Factors for Recurrent UTIs Odds Ratios (ORs) with 95% Confidence Intervals (CI) are reported. Significance level set at p < 0.05. DM: diabetes mellitus, UTI: urinary tract infection

Risk Factor	Odds Ratio (OR)	95% CI	Test Statistic (χ²)	p-value
Female gender	2.84	1.72–4.69	15.27	<0.001
HbA1c >7.5%	3.12	1.98–4.91	18.63	<0.001
DM >10 years	2.45	1.53–3.93	12.11	0.002
Diabetic nephropathy	2.19	1.22–3.93	6.59	0.01
Previous UTI hospitalization	3.67	1.90–7.06	20.74	<0.001

## Discussion

This study evaluated the clinical characteristics, microbial spectrum, and risk factors associated with recurrent urinary tract infections (rUTIs) in patients with diabetes mellitus. The findings confirm that rUTIs are highly prevalent in this population, with more than half of the patients experiencing three or more episodes annually. This highlights a significant disease burden and supports earlier evidence suggesting that diabetic individuals are more susceptible to recurrent infections due to multiple pathophysiological mechanisms. Our results demonstrated a strong association between recurrent UTIs and several clinical factors, including female gender, poor glycemic control (HbA1c >7.5%), longer duration of diabetes (>10 years), and the presence of diabetic nephropathy. These risk factors have also been reported in previous studies and can be attributed to the cumulative effects of metabolic dysregulation, immune dysfunction, and anatomical predispositions inherent to diabetes.

The microbial profile in our study was consistent with global trends, with Escherichia coli being the predominant pathogen (56.3%), generally causing both community- and hospital-acquired urinary tract infections among diabetics [14]. However, the notable presence of non-E. coli uropathogens, such as Klebsiella pneumoniae, Enterococcus faecalis, and Pseudomonas aeruginosa, suggests a shift toward more resistant and healthcare-associated infections, especially in patients with previous hospitalizations. This emphasizes the importance of considering patient history and local epidemiology when formulating empirical treatment strategies [15].

The study revealed an alarmingly high rate of antibiotic resistance among uropathogens. Nearly half - 46% - of the isolates were identified as multidrug-resistant organisms (MDROs), while 36.9% were producers of extended-spectrum beta-lactamases (ESBLs). These are significantly greater than in non-diabetic populations and imply that extensive use of antibiotics, fulsome patient follow-up, highlighting the importance of basing therapy decisions on culture and sensitivity results [16]. The fluoroquinolone sensitivity rate against E. coli was only 36.2%, indicating limited effectiveness; data reflects the global tendency toward resistance and the necessity of revising empirical treatment standards.

On the other hand, the ongoing efficacy of nitrofurantoin and fosfomycin against those pathogens indicates their value as first-line agents for dealing with uncomplicated UTIs in diabetic patients [17]. However, treatment decisions should ideally be guided by culture and sensitivity results to prevent further resistance development. A clear statistical relationship was determined between high hemoglobin A1c values (HbA1c > 7.5%) and recurrent infections (OR 3.12, p < 0.001), supporting previous studies. Rising levels of blood glucose damage the function of neutrophils, weaken the uroepithelial barrier, and lead to glucosuria, thus facilitating bacterial colonization and the development of biofilms [13]. In the study, a history of UTI-related hospitalizations was found to be associated with a three-fold higher risk of resistant infections, documented by an odds ratio of 3.67. This observation means that hospitalization may act as a proxy for severe infection or antibiotic exposure by broad-spectrum antibiotics, thereby indicating a conservative attitude towards prescribing antivirals to these people [18, 19].

Despite the robust data collected, this study has several limitations. The retrospective design inherently carries the risk of incomplete or inconsistent documentation. Important behavioral risk factors such as sexual activity, personal hygiene practices, fluid intake, and medication adherence could not be assessed due to the unavailability of relevant data in medical records. Furthermore, the study population was hospital-based, which may limit the generalizability of findings to the broader diabetic community or primary care settings.

Nevertheless, our findings have significant clinical implications. They reinforce the need for regular monitoring of glycemic control, proactive screening for urinary symptoms in high-risk patients, and prompt culture-based management of UTIs. Non-antibiotic preventive strategies, such as cranberry extract, probiotics, and topical estrogen in postmenopausal women, should be explored further as part of a comprehensive, patient-centered approach. Public health efforts should also prioritize education on UTI prevention, especially among diabetic women and those with long-standing disease.

## Conclusions

It is concluded that recurrent urinary tract infections are highly prevalent among diabetic patients, with poor glycemic control, female gender, longer duration of diabetes, and diabetic nephropathy emerging as significant risk factors. The predominance of Escherichia coli as the causative organism, along with an alarming rise in multidrug-resistant and ESBL-producing pathogens, highlights the urgency of reassessing empirical treatment strategies. The study underscores the importance of strict metabolic control, personalized antibiotic therapy guided by culture sensitivity, and early identification of high-risk individuals to prevent complications. Timely interventions and a multidisciplinary approach are essential to mitigate the burden of recurrent UTIs in the diabetic population and to improve long-term clinical outcomes.
